# Lights and Shadows of TORCH Infection Proteomics

**DOI:** 10.3390/genes11080894

**Published:** 2020-08-05

**Authors:** Janaina Macedo-da-Silva, Claudio Romero Farias Marinho, Giuseppe Palmisano, Livia Rosa-Fernandes

**Affiliations:** 1 Glycoproteomics Laboratory, Department of Parasitology, University of Sao Paulo, Sao Paulo 05508-000, Brazil; janainamace@hotmail.com; 2 Laboratory of Experimental Immunoparasitology, Department of Parasitology, University of Sao Paulo, Sao Paulo 05508-000, Brazil; marinho@usp.br

**Keywords:** TORCH, infections, pregnancy, congenital abnormalities, proteomics, mass spectrometry

## Abstract

Congenital abnormalities cause serious fetal consequences. The term TORCH is used to designate the most common perinatal infections, where: (T) refers to toxoplasmosis, (O) means “others” and includes syphilis, varicella-zoster, parvovirus B19, zika virus (ZIKV), and malaria among others, (R) refers to rubella, (C) relates to cytomegalovirus infection, and (H) to herpes simplex virus infections. Among the main abnormalities identified in neonates exposed to congenital infections are central nervous system (CNS) damage, microcephaly, hearing loss, and ophthalmological impairment, all requiring regular follow-up to monitor its progression. Protein changes such as mutations, post-translational modifications, abundance, structure, and function may indicate a pathological condition before the onset of the first symptoms, allowing early diagnosis and understanding of a particular disease or infection. The term “proteomics” is defined as the science that studies the proteome, which consists of the total protein content of a cell, tissue or organism in a given space and time, including post-translational modifications (PTMs) and interactions between proteins. Currently, quantitative bottom-up proteomic strategies allow rapid and high throughput characterization of complex biological mixtures. Investigating proteome modulation during host–pathogen interaction helps in elucidating the mechanisms of infection and in predicting disease progression. This “molecular battle” between host and pathogen is a key to identify drug targets and diagnostic markers. Here, we conducted a survey on proteomic techniques applied to congenital diseases classified in the terminology “TORCH”, including toxoplasmosis, ZIKV, malaria, syphilis, human immunodeficiency virus (HIV), herpes simplex virus (HSV) and human cytomegalovirus (HCVM). We have highlighted proteins and/or protein complexes actively involved in the infection. Most of the proteomic studies reported have been performed in cell line models, and the evaluation of tissues (brain, muscle, and placenta) and biofluids (plasma, serum and urine) in animal models is still underexplored. Moreover, there are a plethora of studies focusing on the pathogen or the host without considering the triad mother-fetus-pathogen as a dynamic and interconnected system.

## 1. Background

Infections during pregnancy represent a worrying global public health problem [1]. The placenta is a crucial organ for the maternal–fetal interface, being responsible for the embryo oxygen/nutrient supply, maternal immune system regulation, and protection against infectious agents [2]. However, different viruses, bacteria, protozoa, and fungi can cross the transplacental barrier and affect the fetus before birth, thereby impairing intrauterine growth and promoting spontaneous abortion or premature birth [3]. Several pathogens, such as rubella, varicella, toxoplasmosis, and human cytomegalovirus (CVM) have vertical transmission capacities [1]. The last human congenital infection was described in 2015, with in utero exposure to the Zika virus (ZIKV) detected during an outbreak in Brazil [4,5]. The term TORCH is used to designate the most common congenital infections, where: (T) refers to toxoplasmosis, (O) means “others” and includes syphilis, varicella-zoster, parvovirus B19, ZIKV, human immune deficiency virus (HIV), malaria, among others, (R) refers to rubella, (C) to CMV infection, and (H) to herpes simplex virus (HSV) infections [6,7].

**Figure 1:**
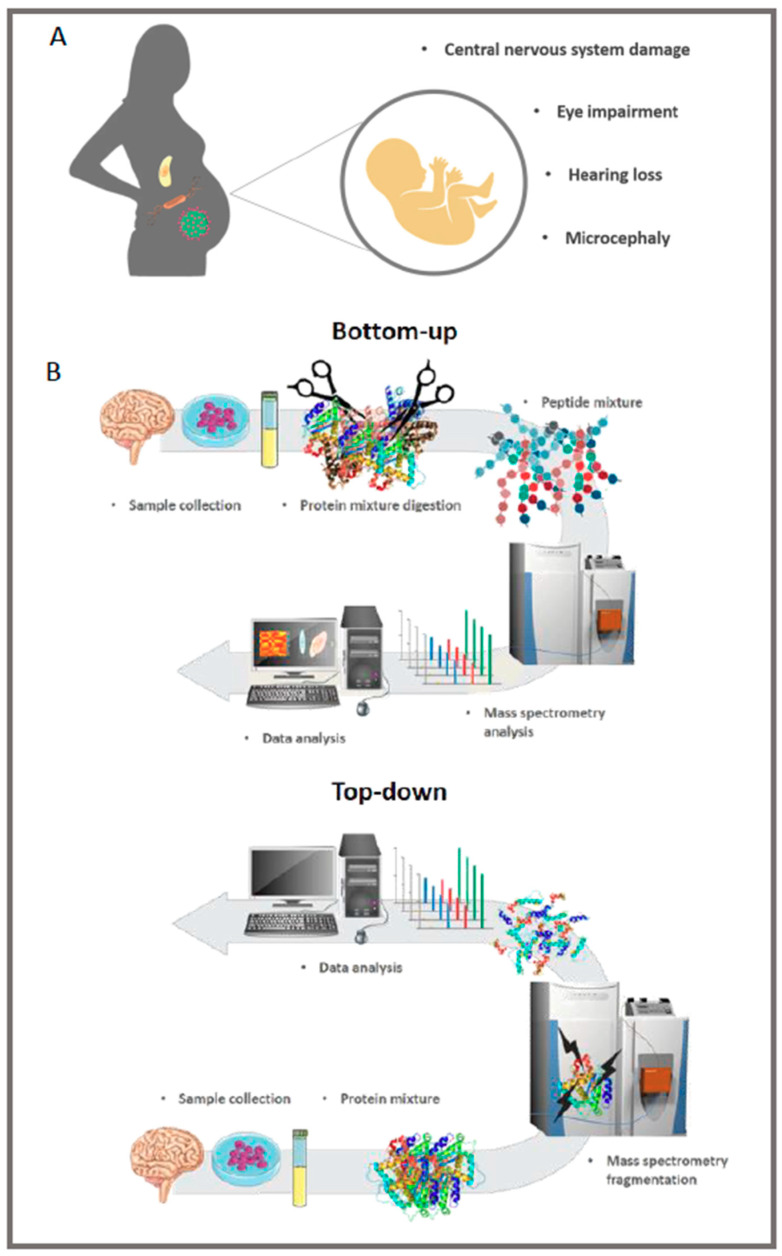
Most frequent symptoms related to TORCH (toxoplasmosis, “others”, rubella, cytomegalovirus infection, and herpes simplex virus infections) diseases. Children can present these symptoms at birth or late, therefore medical follow-up is crucial (**A**). Main stages of bottom-up and top-down proteomic analysis. Different tissues, cell culture or fluids can be analyzed (**B**).

Among the main abnormalities identified in fetuses and neonates exposed to congenital infections are central nervous system (CNS) damage, microcephaly, hearing loss and ophthalmological impairment, all requiring regular follow-up to monitor the phenotype progression (Figure 1A) [3,8]. Specific congenital complications are associated with TORCH infections, as shown in Table 1. Understanding the biology of infections during pregnancy helps in developing therapeutic and/or preventive strategies, especially in epidemic periods, as occurred during the 2015 ZIKV outbreak.

The host–pathogen interaction in congenital infections is a finely dysregulated process that involves the mother–fetus–pathogen triad. Many factors influence the disease progression and severity of infection, such as the mother’s immune status [9]. The placenta is a chimeric organ formed by maternal and fetal parts. The maternal side consists of the uterus stroma, deciduous cells, and immune system cells. The fetal side consists of Hofbauer cells (fetal macrophages), fibroblasts, and the differentiated trophoblast into cytotrophoblasts, syncytiotrophoblasts, and extravillous trophoblast, which is in contact with the maternal side [3]. Studies have shown that trophoblast is permissive for replication of adenovirus [10], CMV [11], ZIKV [12], parvovirus B19 [13], and Hepatitis C virus [14]. In these infectious diseases, high inflammatory responses are required to control the pathogen; however, it can promote complications in pregnancy. Thus, the hormone–immune system relationship is of fundamental importance for the ultimate success of pregnancy and reduction of infection.

During pregnancy, drastic changes occur in progesterone, estradiol, and glucocorticoid levels, which are hormones that directly modulate the immune system [15]. It is evolutionarily advantageous to have reduced inflammatory responses, as these may lead to fetal rejection and increased anti-inflammatory events that stimulate the passive transfer of antibodies from mother to fetus. Exposure to fetal antigens promotes anti-inflammatory responses by the maternal immune system, which results in reduced susceptibility to the development of autoimmune diseases, but directly affects the ability to control infectious diseases [15,16]. Throughout the three trimesters of pregnancy, the pro and anti-inflammatory responses are altered [17]. In the first trimester, low concentrations of estradiol result in increased inflammatory responses mediated by T-helper type 1 (Th1), natural killers (NK), and M1 macrophages; and decreased anti-inflammatory responses, including M2 macrophage activity, T-helper type 2 (Th2), and regulatory T cells. Already in the third-trimester, estradiol levels are increased, resulting in the opposite; that is, the increase of anti-inflammatory responses and reduction of inflammatory responses [18]. Also, there are changes in the levels of different cytokines, such as the reduction of interferon-gamma (IFN-γ) and monocyte chemoattractant protein-1 (MCP-1) and increased levels of tumor necrosis factor alfa (TNF-α) and interleukin-10 (IL10) [19].

Changes in protein abundance, post-translational modifications (PTMs), structure, and function may indicate a pathological condition before the onset of the first symptoms. Moreover, monitoring the levels of these biomolecules may aid in early diagnosis and understanding of a particular disease or infection [20]. Investigation of proteins involved in host–pathogen interaction can shed light on the mechanism of infection [21].

The term “proteomics” is defined as the science that studies the proteome, which consists of the total protein content of a cell, tissue or organism, including PTMs and protein–protein interactions [22]. Currently, bottom-up is the primary strategy to identify and quantify the proteome of a cell, tissue, biofluid, or an organism [23]. However, technological and methodological improvements have boosted the development of middle-down and top-down approaches [24,25,26]. The top-down approach is characterized by the injection of intact proteins in the mass spectrometer, offering approximately 100% of the protein sequence coverage as the main advantage besides the characterization of proteoforms and identification of combinatorial PTMs [24,25]. On the other hand, the bottom-up approach analyzes peptide fragments obtained through enzymatic digestion, such as trypsin cleavage [27]. Liquid chromatography coupled to mass spectrometry (LC-MS/MS) is the method of choice that allows the identification of the protein primary structure, determination of the relative or absolute amount of proteins and interaction between proteins and other biomolecules in complex biological matrices [20,27,28,29]. A schematic workflow of proteomics approaches is shown in Figure 1B. The technique can explore the proteome of tissues, cells, and fluids.

In this review, we discuss the application of proteomic strategies to investigate congenital diseases elicited by the “TORCH” infectious agents, including human cytomegalovirus (HCVM), ZIKV, HIV, HSV, malaria, toxoplasmosis, and syphilis.

## 2. HCMV Is the Leading Cause of Congenital Neurological Disease by Transmission through the Placenta from the Mother to the Child

HCMV is a DNA virus belonging to the *Betaherpesviridae* family, being the largest known human herpesvirus encoding more than 200 conventional reading frames [30]. In healthy adults, HCMV infection is asymptomatic; however, immunocompromised hosts may have fever, night sweats, weight loss, and joint and muscle pain [31]. Congenital transmission of HCMV is the most common in the world, being related to neurodevelopmental delay, hearing loss, microcephaly and psychomotor retardation of neonates [32]. Among live births, seroprevalence is 0.7% in developed countries, while it ranges from 1 to 5% in developing ones [33]. Only 10–15% of neonates clinically manifest symptoms of HCMV infection [34]. Hearing loss may occur at birth or late, with a mean age of 33 and 44 months for symptomatic and asymptomatic babies, respectively. Thus, all infants exposed to HCMV during pregnancy should be followed during the first years of life, regardless of clinical conditions at birth [35]. As HCMV seroprevalence in fertile women varies according to socioeconomic status and age, it is estimated to be 83% in the population, with the highest and lowest marks in the Eastern Mediterranean (90%) and Europe (66%), respectively [36].

Vertical transmission may occur from maternal primary infection during gestation or from reactivation of previous maternal infection [31], as HCMV can be reactivated from latency in bone marrow myeloid cells sporadically [37]. When primary maternal infection occurs in the first trimester, the risk of congenital transmission is 40% and about 25% of infants have complications, whereas, in women with prior immunity, the risk of congenital transmission is 0.2 at 2% [38]. The vertical transmission of HCMV is still not completely understood; however, it is known that infection of the placenta is necessary [39]. Even with limitations, studies have shown responses mediated by antiviral antibodies to structural and non-structural viral proteins in women in the fertile period with previous primary infection [40]. A study that evaluated pregnant women with primary HCMV infection demonstrated that they have higher levels of antiviral antibodies [41]. In addition, the quantification of the anti-viral response in pregnant women with primary infection revealed that those in the group that did not transmit the virus to the fetus had antibodies, including anti-gB, earlier than those who had congenital transmission. Thus, these studies showed the relationship between maternal immunological status and the outcome of vertical transmission [42].

**Figure 2:**
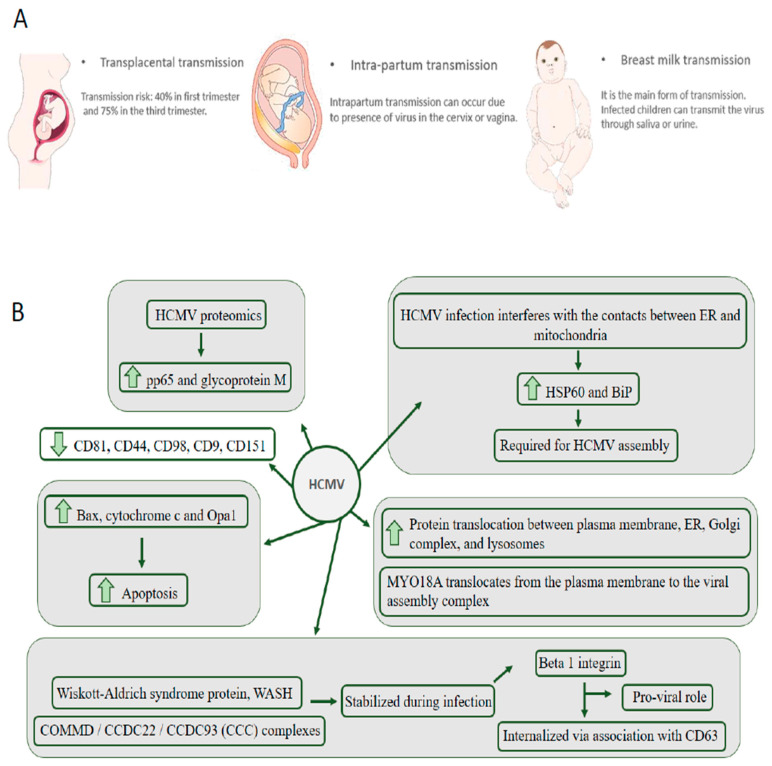
Main forms of maternal–fetal transmission of cytomegalovirus (**A**). Main findings indicated by proteomic studies that explore the proteome of human cytomegalovirus (HCVM) infection (**B**).

Other recurrent forms of viral transmission can be intrapartum and during breastfeeding, as shown in Figure 2A. The most significant epidemiological impact occurs through breast milk transmission, as infected children can transmit the virus through saliva or urine to other children [43]. HCMV has widespread cellular tropism and can infect fibroblasts, macrophages, dendritic cells, hepatocytes, mucous tissue cells, vascular endothelial and epithelial cells, easily enabling systemic infection and transmission between hosts [44]. The most common congenital transmission indicator is the PCR of viral DNA in the amniotic fluid after 21 weeks of gestation. Postpartum detection is done by PCR in other fluids, such as saliva or urine of the newborn.

The first report of the application of proteomic techniques in protein investigation of HCMV virion purified preparations were from Varnum et al., 2004 [45]. An important finding was the identification of more viral proteins than previously known. Among the proteins that showed predominant abundance are the integument protein pp65 and glycoprotein M (gM). In addition, it was possible to identify host cellular proteins associated with the virus, revealing essential data for the understanding of virus ontogeny. Due to the high prevalence and risk associated with infection, the National Vaccine Advisory Committee (NVAC) has added HCMV to the list of high priority infections for vaccine development [46]. Recent advances have shown that the recombinant viral glycoprotein B (gB) vaccine is beneficial in preventing infection in women; however, there is no licensed vaccine to date [47,48]. For vaccine development and targeted therapeutic strategies, understanding the temporal and spatial interactions between the virus and the host cellular machinery is crucial [46]. The study by Viswanathan et al., 2017 [49] provided substantial evidence of virus interaction with the infected cell membrane. The authors used a culture of amino acid stable isotope-tagged fibroblasts (SILAC) infected with HCMV to show that CD44, CD81, CD98, caveolin-1, and catenin delta-1 were downregulated in infected cells. Interestingly, the CD81 protein, a member of the tetraspanin family, was identified downregulated during UV-inactivated virus infection, evidencing the possibility of this protein being involved with viral entry into the host cell. The authors performed experiments to evaluate whether tetraspanin-enriched microdomains (TEM) are required for viral entry and concluded that blocking TEMs inhibits viral entry and correlates with reduced levels of CD9, CD81, and CD151. These data provide strong evidence on the connection between TEMs and the successful HCMV infection, revealing important clues to possible antiviral therapies. In 2018, Martinez-Martin et al., [50] developed a mass spectrometry-based workflow for the detection of receptor–ligand interactions. A library consisting of single transmembrane receptors allowed the identification of neuropilin-2 as the receptor for HCMV infection in epithelial cells among other interactors, which was further characterized by cell-based assays and electron microscopy [50]. The developed framework could also be applied to increase the understanding of other virus-cell interactions.

Successful HCMV infection requires an abundant supply of energy from the infected cell and needs continued cell survival. The high-energy demand induces an increase in glucose and glutamine consumption, which promotes the reprogramming of the metabolic carbon flow in favor of its own metabolic membrane production program for newly assembled viral particles. Also, to prolong cell viability, HCMV inhibits different mitochondrial proapoptotic signaling pathways. These apoptotic activities performed by mitochondria are modulated by contact with mitochondrial associated membranes (MAM), which are subdomains of the endoplasmic reticulum (ER) [51]. The contact between HCMV and MAMs, causes the virus to acquire control over apoptotic events carried out by mitochondria and, thus, can prolong the life of infected cells. To better understand the host cell-virus interaction, Zhang et al., 2011 [52] investigated the MAMs proteome to understand how HCMV infection interferes with the contacts between ER and mitochondria. The authors used forward and reversed SILAC-based quantitative proteomics to evaluate changes in infected and uninfected human foreskin fibroblasts (HFFs) 72 h post-infection. A total of 1719 proteins were identified, of which 991 were identified by at least two unique peptide hits in the forward (unlabeled uninfected cells, SILAC-labeled HCMV infected) and reversed (SILAC-labeled uninfected cells and unlabeled HCMV infected cells). These proteins were associated with microsomes, mitochondria, and cytosol, indicating enrichment of MAMs. The abundance of proteins required for HCMV assembly, such as HSP60 and BiP, were increased in the ER-mitochondria contact region. In addition, apoptosis promoting proteins such as Bax, cytochrome c, and Opa1 were identified upregulated after infection, suggesting a positive feedback mechanism for apoptosis signaling. Overall, the data indicate that HCMV infection alters the MAMs protein pool to increase cell survival and protein translation, benefiting progeny production. A complete analysis of the effect of infection on the organelles of infected fibroblasts was performed by Jean Beltran et al., 2016 [53]. Fluorescence microscopy analysis 24, 48, 72, 96, and 120 h after infection showed mitochondrial network fragmentation from 48 hpi. The proteomic data showed that the total protein distribution of organelles is altered and that 374 proteins presented translocation between organelles after infection, with a higher translocation between plasma membrane, ER, Golgi complex, and lysosomes. Interestingly, myosin (MYO18A) translocates from the plasma membrane to the viral assembly complex, providing significant findings for understanding the biology involved in HCMV infection. Recently, the temporal protein complex dynamics during HCMV infection was monitored using thermal protein profiling combined with mass spectrometry [54]. The HCMV interaction with host cell receptors, HCMV replication, assembly and egress of the virion complex involves the interaction of several host–host, host–viral, and viral–viral protein interactions that are temporally and spatially controlled [55]. Over 200 high confidence protein complexes were monitored and grouped in 8 clusters along the infection. The Wiskott–Aldrich syndrome protein and SCAR homolog (WASH) and the COMMD/CCDC22/CCDC93 (CCC) complexes, involved in cargo sorting and endosomal trafficking, were stabilized during infection suggesting a novel regulatory mechanism during HCMV infection [54]. Integrin beta 1, a known receptor for HCMV, was stabilized during infection. Using microscopy and molecular biology techniques, it was shown that integrin beta 1 is internalized via association to CD63 before degradation, indicating a pro-viral role for CD63. The proteome of HCMV-infected patients was little explored. Liu et al., 2007 [56] evaluated the alteration of serum proteins in 45 children divided between HCMV and hepatitis infected groups (*n* = 20), control (*n* = 5), infected with hepatitis and without HCMV (*n* = 10) and infected with HCMV and without hepatitis (*n* = 10). Serum protein features were detected using WCX2 chips combined with surface-enhanced laser desorption ionization time-of-flight mass spectrometry. The data obtained showed that platelet factor 4/CXCL4 and interleukin-25 (IL-25) are possibly involved in the pathogenesis of HCMV. However, different assays and validation methods are needed to better understand serum proteome alteration in HCMV infection. Figure 2B shows a summary of the main findings of proteomics applied to the investigation of HCMV infection [56].

## 3. ZIKV: In 2015, the World Health Organization Reported Cases of Neurological Disorders in Infants Who Had Their Mothers Exposed to the Virus during Pregnancy

ZIKV is a flavivirus that was first identified in 1947 in Uganda [57], which consists of a single-stranded positive-sense RNA molecule encoding a polyprotein that is cleaved into three structural proteins and seven nonstructural (NS) proteins. Several studies suggest that NS1 is secreted and contributes to the evasion of the host antiviral response [58]. Initially, ZIKV infection was related to flu-like clinical symptoms, including fever, headache, malaise, and rash. However, during an outbreak in French Polynesia in 2013, the infection was associated with Guillain-Barré syndrome and the possibility of mother-to-child transmission [57]. In 2015–2016 a ZIKV epidemic was reported in the Americas, in which about 2700 cases of microcephaly were reported in neonates who had their mothers exposed to ZIKV during pregnancy [59,60]. The pathogens present in the maternal bloodstream are transmitted to the fetus by the placenta, causing an intense inflammatory process [61]. The immunological evaluation of pregnant women with acute ZIKV infection demonstrated a strong response mediated by TCD4+ and TCD8+ cells specific to the virus a few days (2–3 d) after the first symptoms. In addition, there is evidence that previous DENV infection affects the humoral response, resulting in an increased frequency of TCD8+ cells [62,63]. One study examined the impact of pregnancy on innate immune responses during ZIKV infection. The results showed that mice in the control group had a higher frequency of uterine macrophages and tolerogenic dendritic cells, on the other hand, the infected group showed a lower frequency of CD45+IL-12+ and CD11b+IL-12+ cells in the uterus and spleen, demonstrating that maternal immunological changes play an important role in the final outcome of infection and maternal–fetal transmission [64].

Moreover, viral RNA was identified in the brain, placenta, and amniotic fluid of affected babies, reinforcing evidence of congenital transmission [60]. The primary abnormality related to congenital ZIKV infection is microcephaly. Still, other clinical reports include other malformations such as eye abnormalities, hearing loss, high muscle tone, and arthrogryposis, which together characterize congenital Zika syndrome (CZS) [65,66].

According to the World Health Organization (WHO), about 5 to 15% of newborns exposed to ZIKV present complications, with congenital malformations in symptomatic and asymptomatic infections being reported. Moreover, clinical evidence shows that healthy ZIKV-exposed infants can develop severe neurological complications several months after birth [67]. In the US, 6% of babies exposed to ZIKV in utero had congenital disabilities, but when exposed children born without complications were followed up, the number of complications associated with ZIKV increased to 14% [67,68] revealing that the pathogenic mechanisms of long-term infection need to be explored. Neuroimaging tests have shown changes in the brain, such as ventricular enlargement and subcortical calcifications in normocephalic babies exposed to ZIKV [69]. Another study suggests that approximately 60% of exposed babies born without clinical evidence of ZIKV infection have seizures at some stage of development [70]. It is worth noting that babies exposed during the epidemic in the Americas are 3–4 years old, and so far, there are no reports of which symptoms have arisen over time.

ZIKV infection appears to vary in severity depending on the genetic characteristics of the host, as exposure to the virus is associated with mild or severe symptoms. In mice, systemic ZIKV infection has been shown to require a limited interferon-mediated immune response, obtained by eliminating interferon pathway (IFN) members [71], resulting in an ineffective or non-existent response. There is a clear need for comprehensive information on which infection mechanisms result in ZIKV-induced neurological damage. Because the viral replication process manipulates the host’s cellular machinery, the identification of proteins involved in cell invasion may be relevant to the discovery of new antiviral targets. Xin et al., 2017 [72] applied an iTRAQ-based quantitative proteomics approach on *Aedes albopictus* C6/36 infected cells, one of the major vectors of ZIKV spread, and human HeLa cells. A total of 200 differentially regulated proteins were identified, with the upregulated CHCHD2 protein in C6/36 and HeLa cells. Additional experiments have shown that the protein may benefit viral replication and inhibit interferon-beta production in HeLa cells, demonstrating that possibly up-regulation of CHCHD2 is helping viral replication. Another interesting finding was the relationship of the ubiquitin–proteasome system (UPS) to ZIKV entry into the host cell, in which the proteasome 20S portion inhibitor, called bortezomib, inhibited ZIKV infection.

Garcez et al., 2016 [73] combined proteomic approaches to determine proteome variation between mock-infected and ZIKV-infected neurospheres. The results showed a total of 199 downregulated and 259 upregulated proteins between the two biological conditions, including toll-like receptor 4 (TLR4) and RNA Helicase DDX6, which participate in viral recognition. The biological processes related to downregulated proteins in ZIKV infected neurospheres are involved in cell shutdown, regulation and folding of proteins. Another study applied proteomics and phosphoproteomics techniques in the analysis of NPCs and neuronal cell line SK-N-BEB2 infected with ZIKV to identify cellular targets of ZIKV proteins. A total of 386 proteins specifically associated with ZIKV or activities of other flaviviruses have been identified. Interestingly, proteins that play essential roles in neuronal development, retinal defects and infertility have been identified. Perhaps the most interesting data from the work is obtained with phosphoproteomics techniques, which revealed a total of 1216 phosphorylation sites specifically regulated after ZIKV infection, indicating modulation of critical signaling pathways, such as MAPK-ERK and ATM-ATR [74]. Recently, our group [75] applied a proteomic approach to evaluate the alteration of molecular pathways in NPCs and neurons derived from induced pluripotent stem cells infected with the Brazilian strain ZIKV (ZIKV-BR) and African strain MR-766 (ZIKV-AF). The results showed that infection with ZIKV-BR in NPCs results in alteration of pathways related to neurological diseases, cell death, and embryonic survival and development. Besides, it has been shown that HSPB1 protein, involved in neuronal differentiation, has been identified downregulated in infected NPCs. Moreover, infected neurons that were differentiated from NPC showed alterations in neurogenesis and synaptogenesis processes. These data provide important insight into the mechanisms by which the Brazilian ZIKV strain can trigger neuronal abnormalities [75].

A complicating factor in the diagnosis and understanding of ZIKV pathogenesis is its molecular proximity to other flaviviruses, such as dengue virus (DENV). In addition, the circulation areas of these flaviviruses coincide, and many patients have cross-infection. It has been hypothesized that just as an individual is at increased risk of serious illness following infection with a new DENV serotype, previous DENV infection could potentiate ZIKV infection [76]. Allgoewer et al., 2019 [77] evaluated the serum of adult patients with a positive diagnosis of DENV and ZIKV. High-resolution large-scale quantitative proteomics based on data-dependent and data-independent acquisition of 122 sera from a cohort of 62 dengue and Zika virus-infected patients allowed the identification of 300 proteins. Statistical treatment was applied to identify proteins with predictive power to distinguish infection by the two viruses. In total, 26 proteins related to pregnancy and brain were differentially expressed. Of the 26 proteins, those with the greatest predictive power were Platelet Factor 4 Variant 1 (PF4V1), Fibrinogen Alpha (FGA), and Gelsolin (GSN). Song et al., 2018 [78] evaluated the serum of patients infected with ZIKV and DENV using protein arrays and selected a panel of viral proteins capable of distinguishing ZIKV and DENV infections with 90% accuracy, revealing the utility and relevance of the study of proteins involved with the pathogenesis of infections.

## 4. HIV: Vertical Transmission Is the Leading Cause of Infection in Children under 13 Years

Phylogenetic analyses indicate that HIV was introduced into the human population between 1920 and 1940. However, only in the 1980s clinical manifestations of what became known as acquired immunodeficiency syndrome (AIDS) arose as a public health concern. HIV is classified as type 1, HIV-1, which is the most common type in the world, and type 2, HIV-2, which is more prevalent in western Africa and India [79,80]. Studies on the pathogenesis mechanisms have revealed that HIV infects prevalently CD4+ memory T-cells with subsequent depletion and binds to chemokines CXC-chemokine 4 (CXCR4) and CC-chemokine receptor 5 (CCR5) promoting conformational changes that allow entry of the virus in the cell [81]. The first symptoms of infection resemble those of flu such as fever and fatigue, going through a long period without clinical manifestations until it progresses to AIDS, which has symptoms such as weight loss, and recurrent infections. In advanced stages, it has been described that infection can cause severe neurological damage, which may lead patients to mild forgetfulness and frank dementia. Damage to the nervous system is due to neuronal death which occurs as a result of the secretion of TNF-alfa, interleukin 1-beta, inflammatory pathway-inducing chemokines, and viral proteins secreted by infected macrophages and perivascular microglia [82].

**Figure 3:**
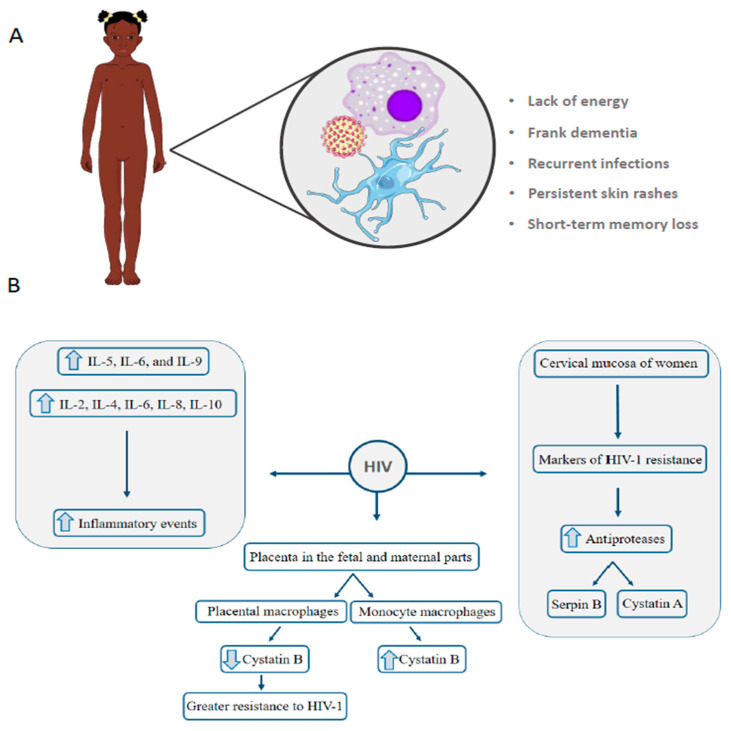
Main symptoms in children who contracted HIV through vertical transmission. Macrophages and microglia are reservoirs for viral multiplication and their immune functions are impaired (**A**). Main findings indicated by proteomic studies that explore the proteome of HIV infection (**B**).

HIV-1 has already been detected in the placenta in the fetal and maternal parts and can to reach fetal circulation through the villous capillaries [83]. Vertical transmission of the virus is the leading cause of HIV-1 infection in children under 13 years (80–90%). It is the only source of transmission in this population in countries with strong blood transfusion regulation and control [83]. The main symptoms observed in children victims of vertical transmission is similar to that found in adults, as shown in Figure 3A.

Maternal–fetal transmission may occur during pregnancy, breastfeeding or childbirth. Infection in children causes more pronounced symptoms and faster progression, resulting in a higher mortality rate [84]. Women who receive antiretroviral treatment have a transmission rate of only 2%, while women who do not receive treatment have a rate of 25%. Interestingly, 75% of the cases in which women do not receive treatment, there is no transmission to the fetus, even with exposure during the gestational period, suggesting that different factors influence the transmission. Higher levels of IL-5, IL-6, and IL-9 interleukins are related to intrauterine but not intrapartum vertical transmission [85]. A study looked at the impact of pregnancy on the T-cell population in HIV-1-positive pregnant women and non-pregnant women. Among pregnant and non-pregnant women, there were changes in the secretion of IFNγ, IL-2, IL-10, and granzyme B in peripheral blood. On the other hand, HIV-1 positive pregnant women showed a decrease in the IL-10 response (anti-inflammatory cytokine) [86]. IL-10 and IL-4 play an important role in protecting the fetus from local or systemic inflammation through inhibition of lymphokine-activated NK [87]. Proteomics was also employed to investigate the mechanisms of altered susceptibility to HIV-1 in female sex workers. In this study, the cervical mucosa of women with HIV-1 resistance was evaluated using 2D-DIGE to determine markers of HIV-1 resistance. More than 15 proteins were differentially regulated between HIV-1-resistant women and controls. Women resistant to the virus have a higher abundance of protease inhibitors, such as serpin B and cystatin A, which is a known anti-HIV-1 factor [88].

Monocytes, tissue macrophages, microglia, and dendritic cells play important immunological functions against infections and studies show that these cells are reservoirs of HIV multiplication, however, each cell type has specificity for viral replication [82]. Since placental monocytes have a greater ability to restrict viral replication compared to monocyte-derived macrophages, Luciano-Montalvo et al., 2008 [89] evaluated the macrophage proteome derived from monocytes and placental macrophages infected or not with HIV. Cystatin B protein (CSTB) has been identified downregulated in uninfected and HIV infected placental macrophages. siRNA silencing assays against CSTB treatment in monocyte-derived macrophages showed a possible association of protein expression with HIV replication, which is aligned with placental macrophages showing higher resistance to HIV-1 infection than monocyte derivatives [89]. Garcia et al., 2009 [90] also assessed the alteration of placental macrophage proteome compared to monocyte-derived macrophages by evaluating the supernatant collected from the culture of these cells. Peroxiredoxin 5 protein, which performs antioxidant and antiviral activities, has been identified as upregulated in the placenta macrophage supernatant. As in the work of Luciano-Montalvo et al., 2008 [89], CSTB levels were lower in the placental macrophage supernatant, providing clues as to how placental secreted proteins may protect the fetus in cases of vertical transmission of HIV-1. Proteomic analysis of urine samples collected from HIV-infected children with HIV-associated nephropathy compared to HIV-infected children with hemolytic uremic syndrome and with no renal diseases was performed using 2DE-MS approach [91]. Higher levels of β2-microglobulin and retinol-binding protein were associated with glomerular and tubular injury. Moreover, higher levels of iron and iron-related proteins, such as hemopexin, transferring, and haptoglobin, were confirmed by ELISA in HIV-infected children with renal diseases. The main findings of the proteomic studies are shown in Figure 3B. It is worth emphasizing the need for further studies applied to HIV, since, to date, the data show interesting insights into the pathogenesis of the disease.

## 5. HSV: Infection in Newborns Can Affect Multiple Organs, Central Nervous System, Eyes, Skin, and Mouth

HSV is a double-stranded DNA virus belonging to the *Herpesviridae* family and has two more aggressive types, HSV-1 and HSV-2 [92]. HSV infections have been known since ancient Greece; however, only in the past 50 years, applied studies have provided information on the pathogenesis of the virus. HSV is prevalent worldwide, does not depend on seasonality and preferentially infects humans [93,94]. For infection to occur, HSV must come into contact with mucous membranes or worn skin. Pathogenesis mechanism is initiated by viral binding to at least three cell membrane receptors, followed by the fusion of its envelope with the plasma membrane and transport the envelope to the nuclear pore, where viral DNA is released [94]. Skin and mucous membranes are the most common sites of HSV infection. However, HSV-1 is more associated with encephalitis and face/mouth infections, while HSV-2 is mainly sexually transmitted and causes a genital infection. Both types can present congenital transmission, which can occur during the peripartum period (85%), postpartum (10%), or, at a lower rate, during the gestational period (5%) [95]. The study by Patel et al., 2019 [96] evaluated human and pregnant mouse samples to assess whether previous maternal immunity influences vertical transmission. Maternal anti-HSV IgG was identified in the umbilical cord, showing that maternal immunity can protect the fetus and reduce HSV maternal–fetal transmission.

**Figure 4:**
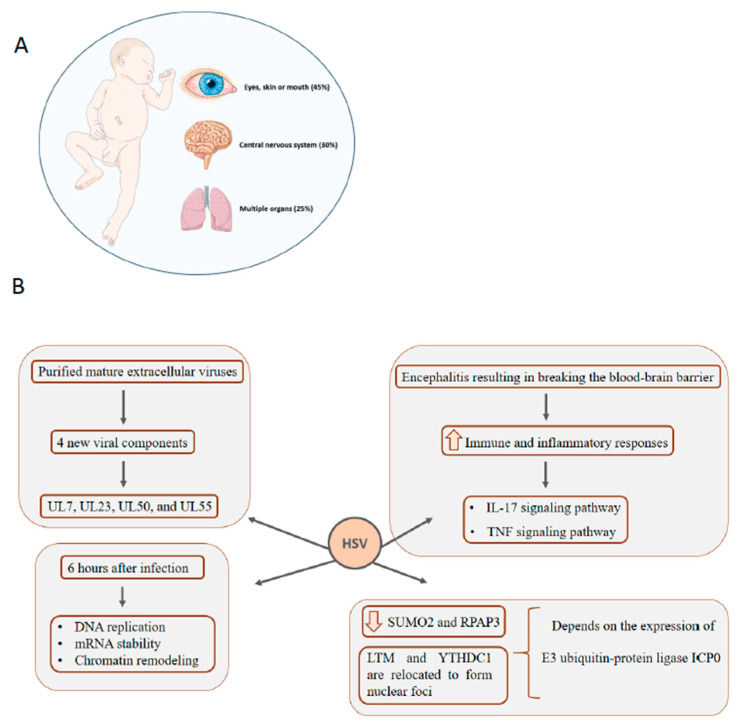
Congenital HSV infection can affect multiple organs, the central nervous system (CNS) and eyes, mouth and skin of newborns. Medical treatment is essential to avoid severe and irreversible damage (**A**). Main findings indicated by proteomic studies that explore the proteome of HSV infection (**B**).

HSV infection in newborns can manifest in three main ways, as shown in Figure 4A: Affecting multiple organs, especially the liver and lungs (25%), affecting the CNS (30%), affecting the eyes, skin or mouth (45%) [97]. Infection of the skin/mouth/eyes usually causes mild damage, but when the disease reaches the CNS, it has a mortality rate of 80% in the absence of treatment [98]. To comprehensively access the HSV-1 proteome, Loret et al., 2008 [99] evaluated by mass spectrometry highly purified mature extracellular viruses. A total of 37 of the 40 known viral components have been identified and, interestingly, four new viral components (UL7, UL23, UL50, and UL55).

Encephalitis resulting from HSV-1 infection usually involves blood-brain barrier rupture, which causes cerebral edema, hemorrhage, leukocyte infiltration, and increased intracranial pressure that can lead to severe brain damage [100]. The molecular mechanisms involved in breaking the blood–brain barrier have been investigated by Liu et al., 2019 [101] using quantitative proteomics to analyze HSV-1 infected mouse brain microvascular endothelial cells bEnd.3 cells. A total of 6761 proteins were identified, with 386 upregulated and 293 downregulated compared to the control. Gene ontology analysis was performed to determine the biological processes in which these differentially expressed proteins act, and the results showed the enrichment of pathways related to defense response, immune system process, and response to external stimulus. The major enriched pathways are involved in immune and inflammatory responses, such as the IL-17 signaling pathway, TNF signaling pathway, and cytokine-cytokine receptor interaction pathway.

The first global comparison of proteome alteration in the early stages of HSV-1 infection was performed by Antrobus et al., 2009 [102]. The authors used human epithelial type 2 (HEp-2) cell line with and without infection with HSV-1 to evaluate proteome alteration 6 hours after infection. Multiple pathway changes were identified, including DNA replication, mRNA stability, and chromatin remodeling, even within the first hours of exposure. Proteome changes of HEK293 cells infected with HSV-1 after 4, 10, and 24 hpi were determined using SILAC quantitative proteomic approach combined with LC-MS/MS. A total of 2178 host proteins were identified at 4 hpi, 2099 at 10 hpi and 1947 at 24 hpi. The study shows that different nuclear and cytosolic functions are altered by the infection over time. At 10 hpi, the enriched nuclear functions include the negative regulation of metabolic processes and DNA repair. In contrast, highlighted cytosolic functions are response to oxidative stress and regulation of apoptosis, which are initiated at 4 hpi. At the latest time point, enriched molecular functions are the regulation of the response to external stimuli, signal transduction linked to the cell surface receptor and assembly of the protein complex [103]. De novo HSV-1 infection has two phases: lytic and latent. The lytic phase is characterized by epithelial cell infection in the contact region, which is where the virus will multiply and release its descendants. The latency phase occurs in host neurons and is characterized by a long silent period and can be reactivated occasionally [104]. To broaden the knowledge on HSV infection dynamics, Kulej et al., 2017 [105] quantified host and viral proteomes, phosphoproteomes, chromatin-bound proteomes, and PTMs on cellular histones across different time points of HSV-1 lytic infection of human foreskin fibroblast cells. Using that approach, the study correlated different stages of viral lytic infection with dynamic changes of virus and host protein levels and PTMs. Importantly, the authors highlight the importance of epigenetic alterations to host chromatin during HSV-1 infection, especially the increase of histone H3 acetylation [105].

Many studies to understand the pathogenesis of HSV are performed in cell culture assays, but little is addressed on how host cell status influences viral infection. Drayman et al., 2017 [106] used a dynamic proteomics-imaging approach tagging 400 host proteins with yellow fluorescent protein and following their dynamics in HSV-1 infected cell culture at single cellular levels. The authors evaluated how cell variability influences viral gene expression. The paper shows an interesting relationship between SUMO2, RPAP3, SLTM, and YTHDC1 proteins and HSV-1 infection. SUMO2 and RPAP3 abundances are reduced when infection occurs, while SLTM and YTHDC1 are relocated to form nuclear foci. Moreover, this observed modulation is dependent on the expression of viral E3 ubiquitin-protein ligase ICP0, suggesting protein SUMOylation as a process to control protein activity, RNA metabolism, gene expression and viral replication [107]. Figure 4B shows a summary of the main data demonstrated by the works discussed above. Congenital HSV transmission has been little explored by proteomic techniques. Studies have been more focused on analyzing the mechanisms regulated during infection, using mainly in vitro cell models to determine the virus–host interaction. The evaluation of the blood proteome, cerebrospinal fluid, cervical–vaginal fluid, or even the placenta, for example, can provide evidence and strategies on vertical transmission, which can benefit affected mothers and children.

## 6. Malaria: Congenital Malaria Is Defined When the Parasite Is Identified in the Peripheral Blood of a Neonate in the First Week of Life

**Figure 5:**
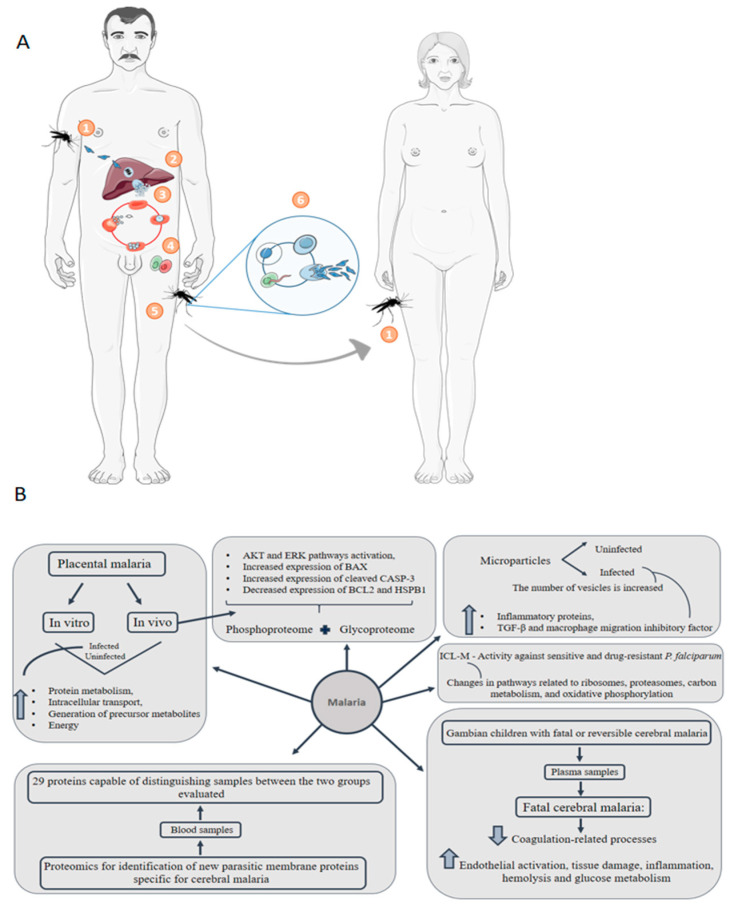
Malaria transmission cycle and representative proteomics-driven studies. (**A**) (1) Protozoa are transmitted to humans in the form of sporozoites through the bite of the female Anopheles mosquito. (2) Sporozoites reach the bloodstream and reach the liver, where they replicate in hepatocytes and mature in schizonts. Schizonts break and release merozoites. (3) Merozoites are capable of infecting erythrocytes, and initiate a cycle of asexual reproduction. Merozoites released in this step can restart a new cycle of asexual reproduction or they can start a cycle of sexual reproduction. (4) In sexual reproduction, male or female gametocytes are formed from merozoites. (5) Gametocytes can be absorbed by mosquitoes during the bite and start a cycle in the insect’s digestive tract. (6) Fertilization occurs that originates the oocyst, which migrates to the mosquito’s hemocele and releases sporozoites, which migrate to the mosquito’s salivary gland. Thus, when biting a new host, the cycle is restarted (1). (**B**) Main results of representative proteomic studies applied to malaria disease.

Malaria is a disease already identified in 91 countries and has more than 120 etiologic agents of the *Plasmodium* genus [108]. Among these, four are capable of infecting human beings; however, *Plasmodium falciparum* and *Plasmodium vivax* are the most prominent. In 2014, 198 million cases of the disease were recorded, with 584,000 deaths [109]. The main regions affected by the disease are Africa, Asia, Oceania, and Central and South America; however, about 90% of deaths from the disease occur in sub-Saharan Africa. Epidemiological data indicate that in high incidence African regions, about 90 to 100% of children under 5 years of age are infected with malaria parasites [110]. Protozoa are transmitted by the bite of the female Anopheles mosquito, so the disease is included in the group of those transmitted by vectors [111]. The success of the infection requires two hosts and the transmission cycle is shown in Figure 5A. The first stage consists of the sting of the infected female, in which the sporozoites are inoculated into the human host. Upon reaching the bloodstream, they infect hepatocytes, where they mature into schizonts, which rupture and release merozoites. After this initial stage of replication called pre-erythrocytic schizogony, which lasts approximately 2 weeks, the parasites begin to multiply asexually in the erythrocytes and start the blood phase of the disease. Briefly, merozoites perform schizogony and evolve into trophozoites, which divide asexually and give rise to a nucleated form called schizonium, which breaks and releases merozoites. Merozoites can start another cycle of asexual reproduction (erythrocytic cycle) or start a cycle of sexual reproduction. In the sexual cycle, the formation of male or female gametocytes occurs, which can be absorbed by mosquitoes during hematophagic feeding. Fertilization occurs in the digestive tract of mosquitoes, which gives rise to the oocyst. During sporulation, the oocyst moves to the mosquito’s hemocele and releases the sporozoites, which migrate to the mosquito’s salivary gland and start the whole cycle again [112].

Infected people usually experience the first symptoms when erythrocyte rupture occurs, including fever, weakness, headache and chills in its uncomplicated form. However, severe forms of malaria are characterized by acute anemia and organ failure, including cerebral malaria, acute respiratory distress syndrome (ARDS) and acute kidney injury [113]. Congenital malaria is defined when the parasite is identified in the peripheral blood of a neonate in the first week of life. The early symptoms demonstrated by infected newborns are fever, anemia, and low birth weight. Other signs and symptoms include jaundice, regurgitation, loose stools, poor diet [114,115] and, in addition, microcephaly and brain damage [116]. Vertical transmission possibly occurs through the sequestration of infected maternal erythrocytes in the placenta intervillous space. When there is an infection, an inflammatory environment with the presence of inflammatory cells and cytokines is created. This environment promotes oxidative stress, which results in placental cell death [117]. Studies indicate that heat shock proteins have been identified in a lower concentration in cells of the infected placenta. Pregnant women are especially vulnerable to malaria infection due to immunological changes during pregnancy and the sequestration of *P. falciparum* infected erythrocytes in the maternal blood spaces of the placenta [118,119]. Proteins from the pathogen are expressed and presented in the human erythrocyte membrane, promoting physiological and morphological changes in the cell [120]. Malaria in pregnancy causes detrimental outcomes for both mother and the developing fetus, who experiences growth restriction and preterm delivery resulting in low birth weight [119,121]. Studies demonstrate the presence of IFN-γ, TNF-α, IL-2, IL-5, IL-13, and IL-10 in the umbilical cord blood of babies exposed in utero to *P. falciparum*, however, if these cytokines are produced by fetal cells or other immune cells has not yet been determined [122]. Recently, inflammasome activation pathways were identified in placental malaria with the production of interleukin-1β (IL-1β) in the infected placenta. Pharmacological treatment using IL-1R antagonist Anakinra improved pregnancy outcomes in an experimental mouse model [123]. These data show that congenital malaria transmission can affect the newborn’s immune system and directly impact the patient’s clinical outcome. Kawahara et.al, 2019 [124] performed a label-based and label-free quantitative proteomic analysis using a *P. berghei*-infected mouse model of placental malaria in parallel to the analysis of placentas collected from pregnant women with past-infection placental malaria. Regulated proteins involved in protein metabolism, intracellular transport, generation of precursor metabolites and energy were among the common enriched pathways in infected mouse and human cohort compared with uninfected ones. Moreover, in the same study, the authors applied a comprehensive TMT-labeled phosphoproteome and glycoproteome analysis of human past-infected placentas and control placentas, showing AKT and ERK pathways activation, increased expression of BAX and cleaved caspase-3 and decreased BCL2 and HSPB1. All the results together feature the occurrence of oxidative stress and apoptotic processes that may be related to adverse fetal outcomes [124]. 

Another complication of *P. falciparum* infection is cerebral malaria. Bertin et al., 2016 [109] applied a proteomic approach to identify new parasitic membrane proteins specific for cerebral malaria. The authors evaluated the alteration of the blood proteome in patients with severe cerebral malaria and uncomplicated malaria. It was possible to identify a group of 29 proteins capable of distinguishing samples between the two groups evaluated, of which 15 and 14 proteins are up and downregulated, respectively, in the group that has severe cerebral malaria. Among the upregulated ones, the 124505939-MESA/PfEMP2 is expressed at the erythrocyte membrane and it is involved in protein trafficking and variant surface antigen export.

In an interesting point of view, a proteomic study evaluated the microparticles content of patients with malaria and healthy controls. Under pathological conditions, including malaria, the number of vesicles is increased. The major finding of the study showed a significant increase in inflammatory proteins such as heat shock proteins, TGF-β and macrophage migration inhibitory factor in microparticles isolated from malaria patients. Moreover, complement systems, hemostasis, hemoglobin subunits and cytoskeletal proteins were significantly upregulated or uniquely present in plasma microparticles isolated from malaria patients [125]. The plasma of 52 Gambian children with fatal or reversible cerebral malaria was assessed by label-free liquid chromatography–tandem mass spectrometry. The study identified 266 differently regulated proteins between the groups evaluated, in which coagulation-related processes were downregulated in the group with fatal malaria and events of endothelial activation, tissue damage, inflammation, hemolysis, and glucose metabolism were upregulated [126].

The identification of *Plasmodium falciparum* surface proteins is of great interest for the development of a vaccine against malaria. However, the high variability of variant antigens (also called erythrocyte membrane protein-1) is a complicating factor; therefore, characterization by mass spectrometry at the protein level is a major challenge [127]. Gonzales Hurtado et al., 2019 [128] developed a pipeline to identify these variant antigens by mass spectrometry. Briefly, the tool searches for high-resolution spectra in a database and an alignment algorithm combines sequences of peptides with the most similar variant antigens, and then calculates a score based on the uniqueness of the peptide used for the inference of the protein. The authors validated the pipeline developed using in vitro assays and analyzing parasite isolates from pregnant women infected with malaria, showing an increase in identified variant antigens.

The development of resistance to antimalarial drugs has been reported, which calls attention to the need for research that seeks the development of new drugs for the treatment of the disease [129]. The compound isocryptolepine, 8-bromo-2-fluoro-5-methyl-5H-indolo [3,2-c] quinoline (ICL-M) showed activity against sensitive and drug-resistant *Plasmodium falciparum*. However, how the ICL-M exerts its antiparasitic function is not well described. Rujimongkon et al., 2019 [130] used proteomics to evaluate the effect of ICL-M on *falciparum*. The authors demonstrated that 112 proteins were differentially regulated after exposure to ICL-M. There were changes in pathways related to ribosomes, proteasomes, carbon metabolism, amino acid biosynthesis, and oxidative phosphorylation. In addition, there was dysregulation of ribosomal proteins after exposure to ICL-M, which was confirmed by electron microscopy images, where loss of ribosomes was observed. This study shows another essential application of proteomics, which is to understand the mechanism of action of drugs. Representative contributions of proteomics to the understanding of malaria disease are shown in Figure 5B.

## 7. Toxoplasmosis: About 75% of Cases of Congenital Toxoplasmosis Have No Clinical Evidence, Making Early Treatment Difficult

*Toxoplasma gondii* is a protozoan responsible for the transmission of one of the most common zoonosis worldwide [131]. Data indicate that about a third of the population has already been infected by the pathogen [132]. When it affects immunocompetent individuals, few or no symptoms are noticed, but immunocompromised individuals may experience flu-like symptoms. Transplanted patients or those with AIDS can have more severe consequences, such as toxoplasmic encephalitis, which can be fatal if left untreated [133].

Vertical transmission of toxoplasmosis parasite can have different clinical manifestations, from miscarriages, premature births, abnormally low birth weights or severe congenital birth defects [134,135]. The mother’s immunity affects the rate of vertical transmission and the severity of the newborn’s clinical manifestations. Analysis of peripheral blood mononuclear cells and IgG1, IgG2, IgG3, IgG4, and IgA antibodies in the serum of pregnant women infected with *T. gondii* revealed that IgG2–4 and IgA antibodies and CD4+, CD8+, and CD19 lymphocytes have higher levels in mothers who have congenital transmission compared to those who do not [136]. When it is possible to identify clinical manifestations, children present fever, hydrocephalus, microcephaly, cerebral calcifications, seizures, and skin rash. Also, they may have epilepsy or deafness months or years after birth [137]. Contrary to most congenital diseases, toxoplasmosis offers a higher risk to the fetus if it is transmitted in the third trimester, where the chance of vertical infection is 60%, while during the first and second trimester this chance is 15–20% [138].

**Figure 6:**
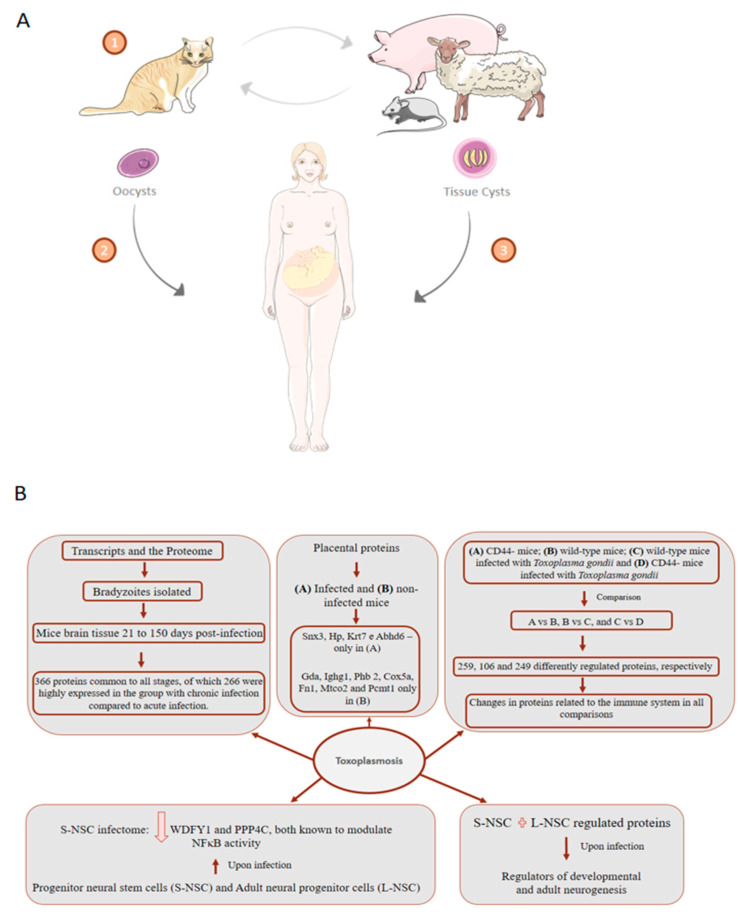
Toxoplasmosis transmission cycle and representative proteomics-driven studies. (**A**) (1) In the cat, after ingesting tissues containing oocysts or cysts, these are released into the body and penetrate the intestinal epithelium where they undergo asexual reproduction followed by sexual reproduction, transforming into oocysts and can be excreted together with the feces. Oocysts can survive for months in the environment and are resistant to disinfectants, freezing, and drying, but destroyed by heating at 70 °C for 10 min. Other animals, such as pigs, sheep, rats, and including man (2) (intermediate hosts), can consume oocysts present in the environment and become contaminated. The oocyst ruptures in the intestine of the intermediate host, releasing the sporozoites that invade the enterocytes. In the enterocyte, each parasite is called a tachyzoite. Tachyzoites spread through the animal’s body and can form cysts in nervous and muscular tissue, which can be consumed by humans and cause infection (3). Tachyzoite multiplies asexually and disrupts the host cell. After the invasion of a new cell by a tachyzoite, the asexual cycle can lead to the formation of intracellular bradyzoites. The formation of bradyzoites begins to occur with greater intensity when the intermediate host develops specific immunity. (**B**) Main findings indicated by proteomic studies that explore the proteome of toxoplasmosis infection.

*Toxoplasma gondii* is an intracellular pathogen found in the forms of oocyst, tissue cyst and tachyzoite. Oocysts are produced in the intestines of cats and can be released in the feces and then contaminate the soil. Tachyzoites are capable of infecting nucleated cells and multiplying in their vacuoles [139]. *Toxoplasma gondii* can cause chronic infection in the brain of its hosts, by passing the morphology of the tachyzoites to neuronal cysts, from which bradyzoites are released. Figure 6A shows a summary of the transmission of *Toxoplasma gondii* between different hosts, including humans. To better understand the effects of *T. gondii* on the brain, Ngô et al., 2017 [140] used a multiplexed isobaric tandem mass tag (iTRAQ) approach to evaluate the proteome upon infection of two humans in vitro models: Progenitor neural stem cells (S-NSC) and adult neural progenitor cells (L-NSC). Analysis of S-NSC infectome showed downregulation of WDFY1 and PPP4C, both known to modulate NFκB activity. WDFY1 induces TLR3 and TLR4 activation of NFκB, the production of type I interferons and inflammatory cytokines [141], compromising the response to parasite infections. When combining S- and L-NSC regulated protein upon infection, up-stream regulators of developmental and adult neurogenesis were identified, such as TGF-β, ERK genes, PI3K, FoxOs, and GM-CSF. Noteworthy, PI3K/Akt is involved in the regulation of endogenous ROS levels during neurogenesis [142]. Interestingly, Ingenuity pathway disease-functions analysis of brain–parasite interactions showed an association with epilepsy, movement disorders, Alzheimer’s disease, and cancer [140].

*T. gondii* bradyzoites have a wall rich in carbohydrates and a slow replication cycle, which allows them to escape from the host immune response within neuronal cysts [143]. One studied analyzed the transcripts and the proteome of bradyzoites isolated from the mice brain tissue 21 to 150 d post-infection. The study identified 366 proteins common to all stages, of which 266 were highly expressed in the group with chronic infection compared to acute infection, including the known bradyzoite markers BAG1, ENO1, and LDH2 [144]. A proteomic study used iTRAQ tagging to track proteins related to CD44, which plays an essential role during infection with *T. gondii* in the mice brain. The experimental design included 4 groups: (A) CD44- mice; (B) wild-type mice; (C) wild-type mice infected with *Toxoplasma gondii* and (D) CD44- mice infected with *Toxoplasma gondii*. Comparisons were made between groups A vs. B, B vs. C, and C vs. D, which showed 259, 106, and 249 differently regulated proteins, respectively. The results showed that there were changes in proteins related to the immune system in all comparisons [145].

To address the role of placenta in abnormal pregnant outcomes caused by *T. gondii* infection, label-free quantitative liquid chromatography-tandem mass spectrometry workflow was used to analyze placental proteins from infected and non-infected mice [146]. A total of 792 proteins were detected, from which Snx3, Hp, Krt7, and Abhd6 were identified only in infected placentas while Gda, Ighg1, Phb 2, Cox5a, Fn1, Mtco2, and Pcmt1 only in uninfected ones. Among the 58 proteins found regulated in placenta tissue upon *T. gondii* infection. Among those, C3, Fga, Plg, and Serpinc1 are implicated in complement regulation and coagulation pathways. Moreover, proteins involved in acetylation, duplication and secretion were also found regulated [146]. In the following year, the function of the immune system in the maternal–fetal interface was explored using tandem mass tag proteomics to quantify human decidual immune proteome upon *T. gondii* infection. Among those the 181 proteins found differentially expressed, 111 were down-regulated in infected cells, including IL1β, MCMBP, COX2, NCBP2-A, HMGN2, ICAM-1, C/EBPβ, Granzyme A, and PAI-2. Resulting functional enrichment analysis showed regulated proteins are involved in several physiological processes of pregnancy, including trophoblast invasion, uterine vascular remodeling, decidualization, embryo implantation, and intrauterine growth [146]. Together, those findings offer insights into the underlying molecular mechanisms for placental involvement in abnormal pregnancy outcomes associated with *T. gondii* infection.

Some drugs used to treat *Toxoplasma gondii* infection and other parasites target mitochondrial proteins [147]. Genomics-based studies have identified homologous genes that encode mitochondrial proteins from other eukaryotes [148]. Because of the critical function of this organelle for infection with *Toxoplasma gondii*, Seidi et al., 2018 [149] characterized the parasite’s mitochondrial proteome and identified 400 proteins, with the majority not being homologous for any of the species infected with *Toxoplasma gondii*. In addition, many of these proteins are crucial to the growth of the parasite. The study also highlights the identification of many proteins that are components of cytochrome c oxidase complex (COX).

Different strains of *Toxoplasma gondii* have already been identified, and it is possible to observe three types, I, II, and III, which are more frequent in North America and Europe. However, in South America, these three strains are sporadically isolated [150]. Little is known about the variation in the proteome of the different strains of the parasite. Thus, Zhou et al., 2017 [151] explored the oocyst proteome of two strains, with more and less virulent phenotypes of *Toxoplasma gondii*. The study identified 374 proteins differently regulated between strains, but the most interesting finding of the study was that of 22 virulence-related proteins, 13 were upregulated in the most virulent strain, and only 2 in the least virulent strain. Another study evaluated the proteome of the three morphological stages of *Toxoplasma gondii* (tachyzoites (T), cyst stages containing bradyzoites (C) and oocysts (O)). The comparison showed a total of 875, 656 and 538 proteins differently expressed in O vs. T, T vs. C, and C vs. O, respectively. The study identified 79 ribosomal proteins, of which 33 and 46 were upregulated in oocysts and cysts, respectively, compared to tachyzoites. The authors suggest that these findings may be related to greater environmental adaptation and escape from the mechanisms of oocysts and cysts, respectively. Representative findings of proteomic studies applied to toxoplasmosis are summarized in Figure 6B.

## 8. Syphilis: Congenital Syphilis Presents about One Million Cases per Year and Is Responsible for more than 300 Thousand Perinatal Deaths

The *Treponema pallidum* is the etiological agent of syphilis, a bacterial disease that presents about 6 million new cases per year worldwide [152]. The majority of new cases related to the disease occur in underdeveloped or developing countries, with an increase of 4000% in new cases only in the last decade in Brazil [153]. The disease has four stages of development, called primary, secondary, latent, and tertiary, which manifest for approximately 10 years. The first clinical symptoms appear three weeks after the local infiltration of *Treponema pallidum* through the subcutaneous tissues. Individuals have ulcerations and multiple injuries to the genitals and other parts of the body involved in sexual contact. After 6–8 weeks, secondary clinical manifestations are observed, such as fever, headache and rashes on the arms, back and especially on the hands, and soles of the feet. After these symptoms, the disease enters the latency phase and can last for years without manifestation. However, individuals are still considered infectious in the first two years. If treatment is neglected, the disease may progress to the tertiary stage, which includes severe symptoms, such as destructive cardiac and neurological conditions [154].

**Figure 7:**
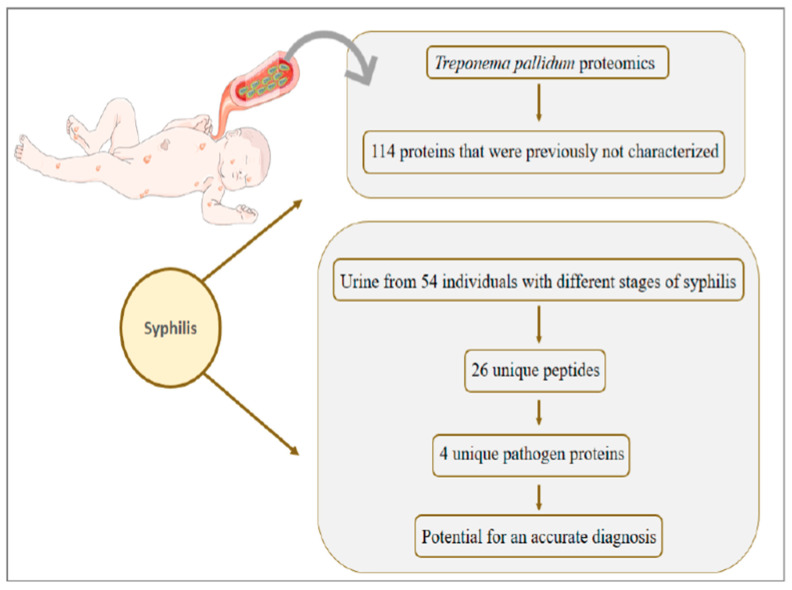
In congenital syphilis, unlike infection in adults, *Treponema pallidum* is released into the fetus bloodstream and quickly reaches multiple organs. The main clinical symptom associated with disease in neonates is skin rashes, which spread throughout the body region. The main findings of the few studies that explore the proteome of syphilis infection are shown.

Congenital syphilis presents about one million cases per year and is responsible for more than 300 thousand perinatal deaths [155]. In the 1990s there was a decrease in the number of cases; however, between 2012–2014 the scenario was reversed and only in the United States the number of cases went from 334 to 458, to reach 918 cases exclusively in 2017 [156,157]. Unlike syphilis in adults, in congenital transmission, *Treponema pallidum* is released into the fetus’ bloodstream and quickly reaches several organs (Figure 7), such as kidney, heart and bones [158]. The immunomodulatory role played by the mother and the fetus in congenital syphilis lacks information. There are few reports in the literature of immunological changes in congenital syphilis, however, one study evaluated neonates after intrauterine infection. The results demonstrated that the levels of IgM, B cells, and immune complexes are increased in infected neonates [159].

Most neonates affected by congenital syphilis have no symptoms, and it is possible to observe the first manifestations only after 3 months of age. The main symptoms include hepatomegaly, jaundice, rhinitis, and skin rashes. But more serious complications can occur if the diagnosis of congenital syphilis is not early, such as hearing loss, secondary glaucoma, scarring of the cornea, and cranial nerve palsy [160].

To our knowledge, proteomic studies applied to congenital syphilis are not present in the literature; however, the mass spectrometry characterization of *Treponema pallidum* was performed by Osbak et al., 2016 [161], in which a total of 557 proteins were identified. A significant finding of the work was the identification of 114 proteins that were previously hypothetical or not characterized. To better understand the infection mechanism of *Treponema pallidum*, a structural bioinformatics approach was applied to better understand the parasite’s protein functions. The study indicates that the tertiary structure modeling generated highly reliable predictions for 80% of the proteome. In addition, modeling based on the tertiary structure noted the same function as pipelines based on primary structure. Furthermore, of 175 proteins that were modeled with high confidence in the study, 167 received functions in the proteome, which was not made in any previous studies.

The diagnosis of the disease is made mainly by serological tests, but the diversity of clinical manifestations and the impossibility of cultivating the *Treponema pallidum* make it impossible to have an ideal alternative test [162]. Seeking an accurate diagnosis that detects the pathogen quickly and non-invasively, Osbak et al., 2018 [163] searched for *Treponema pallidum* antigens in the urine of 54 individuals with different stages of syphilis. The authors identified 26 unique peptides that correspond to 4 unique proteins of the pathogen, which have the potential for an accurate diagnosis of syphilis.

## 9. Congenital Transmission of Varicella, Rubella, and Parvovirus B19 Has a Gap in Proteomic Studies

The proteomic investigation of congenital rubella, varicella and parvovirus B19 infection has been little explored so far, therefore, we emphasize that in vivo and in vitro studies can be performed in order to elucidate the mechanisms involved in the pathogenesis of infections, which are related to different fetal complications. Rubella is a single-stranded RNA virus that occurs worldwide with a seasonal distribution [164]. Congenital rubella transmission is a major public health problem, accounting for more than 100,000 cases annually worldwide [165]. In addition, vertical infection is one of the few known causes of autism. Among the main abnormalities associated with congenital rubella infection, cardiac defects, and deafness stand out. However, there are other related symptoms, such as microcephaly, behavioral disorders, mental retardation, cataracts, and pigmentary retinopathy [166]. Varicella-zoster (VZV) or human herpesvirus type 3, is an RNA virus belonging to the *Herpetoviridae* family and one of the eight types of herpes viruses that infect humans and vertebrates [167]. Congenital chickenpox is rarely transmitted and it is estimated that it affects about 0.5 to 6.5% of newborns [168]. The congenital syndrome is characterized when the pregnant woman becomes infected between 0 and 20 weeks and among the symptoms presented by neonates, it is possible to include skin lesions, muscle atrophy, intrauterine growth restriction, and sensory deficit [169]. Human parvovirus B19 is a DNA virus that presents a 30% risk of maternal–fetal transmission [170]. Parvovirus is a global agent and IgG antibodies against the agent are estimated to be present in 2–15% in the population of children aged 1–5 years; 15–60% in children aged 6 to 19 years and 30 to 60% in adults [171]. Neonates infected by vertical transmission can develop congenital anemia, heart failure, and myocarditis [172].

## 10. Critical Points of Proteomics Approaches Applied to Congenital Diseases

The data shown in Table 2 reveal that different matrices were analyzed by proteomic techniques; however, the vast majority of studies were applied in vitro models. This type of approach provides interesting data on the mechanisms of infection by different pathogens. Besides, it allows the temporal evaluation of the change in the proteome in the cases of infection, for which in vivo models are less effective. Studies that combined proteomic and microscopic techniques were able to evaluate protein translocation after infection, providing essential data for possible drug intervention. When purified viral particles were investigated, unreported proteins were identified, providing a more accurate knowledge of the organism. The placenta is an essential organ in vertical transmission, however, our review shows that this tissue has been little explored by proteomic techniques in vitro or animal models in vertical transmission by HCMV, ZIKV, HSV, and syphilis.

One of the great potential of proteomics is its clinical application, mainly by the identification of biomarkers, which can be defined as biochemical, immunological or genetic variables whose presence is indicative of some phenomenon, such as disease, infection or environmental exposure. These biomolecules have three strands of applications, being indicated as predictive biomarkers when they can offer information about the risks of an individual to contract a disease; diagnostic biomarkers, when able to identify the occurrence of a disease, and prognostic biomarkers when indicate how a disease can evolve [173]. Many pathologies are studied based on the identification of biomarkers, such as cancer, Alzheimer’s, genetic diseases in general and infectious diseases such as leishmaniasis and Chagas disease [174,175,176]. However, our review shows that congenital diseases are being neglected from this perspective, with few studies that seek to explore this field.

Malaria was the disease with the highest number of assessments in human tissues and fluids. On the other hand, HSV is studied mainly by the use of in vitro models. We know that access to patients’ tissues and fluids is a major obstacle to the interaction between clinical medicine and research, but, given the potential that proteomics offers, especially for non-invasive diagnosis, this barrier should be broken or facilitated. In general, this review warns of a gap in the investigation of congenital transmission from a proteomic perspective. Maternal–fetal transmission results in complications that remain untreated, especially for patients living in low-income countries. In addition, these complications constitute an important issue for the public health systems reducing substantially the quality of life of those affected.

## 11. Concluding Remarks

Maternal–fetal transmission significantly reduces the quality of life of affected people, mainly due to the abnormalities presented by neonates and the need for regular medical monitoring. The molecular mechanisms involved in infection are key processes for understanding transmission. Hypothesis-driven approaches to investigate maternal–fetal–pathogen interaction can often be reductionist as they lack the complexity layer to model the multi-dependent nature of these dynamics, which in turn, limits the knowledge that can actually be translated into the development of new prophylactic, diagnostic, and therapeutic procedures.

With the development of new methodologies, instrumentation and analytical tools, mass spectrometry-based proteomics have been applied to investigate different types of hypotheses related to vertical transmission, and the results obtained suggest that multiple proteins or protein complexes actively participate in the success of the infection, enabling the approach. Until now, most of these proteomic studies are performed in cell line models, and the evaluation of human tissues and fluids or even animal models are still little explored. Nevertheless, it is evident the great scientific contribution of the techniques in providing biological data that contribute to the determination of biological models that elucidate the host–pathogen interaction. In addition to this approach focused on the pathological mechanisms, proteomics can be explored in the field of clinical diagnosis involving biomarkers. Although the literature shows few approaches in this field with regard to congenital diseases, it is pertinent to emphasize that benefits, such as early detection, would be useful for a targeted medical intervention that is consistent with the clinical condition of the patient, and thus help to improve quality-affected families’ lives.

## Figures and Tables

**Table 1:** Main symptoms identified in neonates exposed to congenital infections. The + sign means that the indicated symptom has already been related to the disease; while the sign - means that the symptom has little or no relation. Zika virus (ZIKV), human immunodeficiency virus (HIV), human cytomegalovirus (CVM), herpes simplex virus (HSV).

Disease	Intracranial Calcifications	Hearing Loss	Eye Impairment	Microcephaly	Bone Lesions	CNS Damage
**Toxoplasmosis**	+	-	+	+	-	+
**Syphilis**	-	-	-	+	+	+
**ZIKV**	+	+	+	+	+	+
**HIV**	-	-	-	-	+	+
**Varicella**	-	-	+	-	-	+
**CVM**	+	+	+	+	-	+
**HSV**	-	+	+	+	+	+
**Rubella**	+	+	+	+	-	+

**Table 2:** Representative proteomics-driven studies on TORCH associated infections. Examples on biological matrices analyzed, the proteomic strategy and the number of total protein identifications are reported.

Disease	Matrix	MS Approach	Total Identifications	Reference
**HCMV**	Human serum	Label-free quantification with SELDI-TOF-MS	Not available	[56]
	primary human fetal foreskin fibroblasts	TMT quantification and LC-MS/MS on Orbitrap Elite and Fusion	>8000 cellular proteins and 139 canonical and 14 ORFs viral proteins	[177]
	ARPE-19 and Expi293F cells	Easy nLC 1000 HPLC system coupled to an Orbitrap Elite mass spectrometer	1297	[50]
	Purified HCMV AD169 virions	Label-free quantification on a Finnigan LCQ ion trap MS	59	[45]
	MRC5 human lung fibroblasts	Label-free quantification and TMT labeling on a LTQ-Orbitrap XL	4000 host and 100 viral proteins	[53]
	HFFs cells	SILAC labeling with 2D–LC-MS/MS (MudPIT) on a LCQ Deca XP Plus mass	504	[49]
	HFFs cells	SILAC labeling with LC-MS/MS on a LTQ Orbitrap	1719	[52]
	MRC5 cells	TMT labeling with nLC-MS/MS on a Q-Exactive HF	5300	[54]
**ZIKV**	HeLa and HFFs cells	iTRAQ labeling with LC–MS/MS on a TripleTOF 5600	3544	[72]
	NPCs and iPSCs	TMT labeling with nLC-MS/MS on a Q-Exactive HF-Hybrid Quadrupole-Orbitrap	6080	[75]
	Neurospheres	Label-free quantification on a 2D-RP/RP Synapt G2-Si mass spectrometer	Not available	[73]
	NPCs and SK-N-BEB2 cell line	Label-free quantification with AP–LC–MS/MS on a LTQ-Orbitrap XL and Orbitrap Q Exactive HF	386 ZIKV-interacting proteins and 1216 phosphorylation sites	[74]
	Human serum	Label-free quantification with EASY-nLC 1000 on a Q Exactive High	300	[77]
**HIV**	Vaginal discharge	Label-free quantification with 2D-DIGE Nanoflow LC/MSMS on a QStar XL Qq-TOF	72 protein spots with change in volume	[88]
	Monocytes and placental macrophages	Label-free quantification with SELDI-TOF and (LC MS/MS)	Not available	[89]
	Placenta	Label-free quantification with LC–MS/MS on a LTQ XL	Not available	[90]
**HSV**	Purified virions	Label-free quantification with ESI-MS/MS on a QTRAP 4000 linear ion trap mass spectrometer	37	[99]
	HEp-2 cells line	Label-free quantification with 2-DE and LC-MS/MS on a Q-TOF 1 Mass Spectrometer	103 protein spot changes	[102]
	HEK293 cells	SILAC labeling with LC-MS/MS on a Q-Star Elite mass	At 4 hpi, 2178; At 24 hpi, 1947; At 10 hpi, 2099	[103]
	HFF cells	Label-free quantification with LC-MS/MS on a Orbitrap Fusion Tribrid mass spectrometer	4000	[105]
	bEnd.3 cells	TMT labeling with nanoLC-MS/MS on a Q-Exactive Orbitrap	6761	[101]
**Malaria**	Human blood	Label-free quantification with LC-MS/MS on a Linear Trap Quadrupole-Orbitrap Velos	1527	[109]
	Human plasma	Label-free quantification with 2D LC-MS on a LTQ ion trap	1806	[125]
	Human plasma	Label-free quantification with Nano-LC–MS/MS on a LTQ-Orbitrap Velos	504	[126]
	Human blood	Label-free quantification on a LTQ Orbitrap Velos	Not available	[128]
	Infected placentas	TMT labeling with nano-LC-MS/MS on a Orbitrap Fusion	2946	[124]
	Human erythrocytes cell culture	Label-free quantification on a micrOTOF-Q	668	[130]
**Toxoplasmosis**	Cysts from brain and muscle tissues of pigs	iTRAQ labeling with LC–MS/MS on a Q Exactive Orbitrap	2551	[151]
	Primary, neuronal and monocytic stem cells	iTRAQ labeling with LC/MS/MS on a LTQ Orbitrap Velos	4367	[140]
	Brain mice	iTRAQ labeling with 2D-LC-MS/MS on a Orbitrap LC-MS	2612	[145]
	Brain mice	Label-free quantification with LC-MS/MS on a Q-IT-OT Fusion Lumos	1683	[144]
	*T. gondii*-infected and -uninfected placentas of pregnant mice	Label-free quantification on a Q-Exactive Plus Orbitrap mass	792	[146]
	Mitochondria from parasites	Label-free quantification on a Q-Exactive Orbitrap	400	[149]
**Syphilis**	Urine	Label-free quantification on a 2D-LC-MALDI TOF/TOF and LC/ESI-IM-Q-TOF/HDMS	Not available	[163]
	DAL-1 strain bacteria isolated from rabbits	Label-free quantification on a MALDI-TOF/TOF and ESI-LTQ-Orbitrap	557	[161]
